# Treatment of Severe (Stage III and IV) Chronic Pressure Ulcers Using Pulsed Radio Frequency Energy in a Quadriplegic Patient

**Published:** 2008-10-14

**Authors:** Eugene G. Porreca, Gina M. Giordano-Jablon

**Affiliations:** Progressive Hospital, Las Vegas, Nevada

## Abstract

**Objective:** To report an adjuvant treatment to basic wound care of stage III and IV pressure ulcers in a patient with quadriplegia. **Methods:** Pulsed radio frequency energy was used as an adjunct to basic wound care of 3 large, long-standing (6 years) stage III and IV pressure ulcers that were unresponsive to conventional therapy in a 59-year-old man with quadriplegia. **Results:** The ulcers (on right foot, left heel, and sacrum) markedly decreased in size (16.7, 28.5, and 13.1 mm^2^ per day, respectively). The ulcer on the right foot healed within 4 weeks, the left heel ulcer reduced in size by 95% at 7 months, and the large sacral ulcer healed to closure in 11 months. **Conclusion:** Pulsed radio frequency energy treatment with basic wound care, if administered early in the course of pressure ulcer therapy, might avoid the lengthy hospitalizations and repeated surgical procedures necessary for treatment of uncontrolled ulcers, reducing the overall cost of treatment and improving the quality of life for chronically ill or injured patients.

Chronic pressure ulcers are a significant health problem, especially in elderly individuals and individuals with spinal cord injury or other debilitating illness that render them immobile. Chronic open wounds often require many months of treatment and reduce the quality of life of already ill patients. Traditional treatment modalities of pressure ulcers include the use of support surfaces, position changes, nutrition balancing, frequent dressing changes, hydrotherapy, surgical debridement, and surgical reconstruction. Progress generally is slow and often incomplete. All too often amputation becomes the only alternative.

Pressure ulcers present an enormous financial burden for the healthcare system as well as for patients. Pelham et al^1^ noted that although specific costs associated with pressure ulcers are difficult to determine because of comorbidities in chronically ill individuals, in-hospital care of patients with pressure ulcers costs 50% more than care for patients without ulcers. Hirshberg et al^2^ reported an average hospital charge of $48,934 per patient for treatment of ulcers, which did not include the treatment before hospitalization or the cost of pharmaceuticals. Other studies estimate treatment costs in the billions of dollars per year in the United States.^3^,^4^ A need exists for an effective treatment that decreases the healing time and severity of ulcers, is easy to use, and is cost-efficient.

We report a case of a quadriplegic patient with 3 large, long-standing (years) stage~III and IV pressure ulcers that were treated with pulsed radio frequency energy (PRFE).^5^ Previous in vitro studies have suggested that this system may be beneficial in the reparative process of chronic wounds.^5^,^6^

## METHODS

### Case

A 59-year-old man with quadriplegia (C2 fracture), following an on-the-job injury 14 years earlier, presented to the hospital with 3 large, long-standing (6 years) stage III and IV pressure ulcers that were unresponsive to conventional wound care therapy: a 60-cm^2^ left heel ulcer; a 5-cm^2^ right lateral foot ulcer; and a large, 295-cm^2^ presacral ulcer complicated by prior (treated) sacral osteomyelitis. The patient's history was remarkable for diabetes mellitus, respiratory failure, multidrug resistant pneumonia, hypertension, deep vein thrombosis, septic shock, neurogenic bladder, clostridium difficile colitis, and dyslipidemia. Over the course of 6 years of pressure ulcer management, the patient had been treated unsuccessfully with off-loading, frequent and extensive surgical debridement, topical therapy involving hydrogels and alginates, and placement of a rotation flap. Despite his complex medical history, the patient maintained good glucose control through most of this period.

Pulsed radio frequency energy treatment was instituted to each ulcer site as an adjunct to the patient's ongoing basic wound care (periodic wound debridement with enzymatic debriders and application of silver-based dressings on a daily basis). The patient was placed in a comfortable position and the applicator pad was placed directly adjacent to the patient's dressed wound. Pulsed radio frequency energy treatment was delivered through a solid-state, 27.12-MHz, fixed power output radio frequency generator (Provant Wound Therapy System, Regenesis Biomedical, Inc, Scottsdale, Ariz), transmitting a fixed dose of nonionizing, nonthermal radio frequency energy for 30 minutes twice daily, every 8 to 12 hours.

## RESULTS

**Figure 1:**
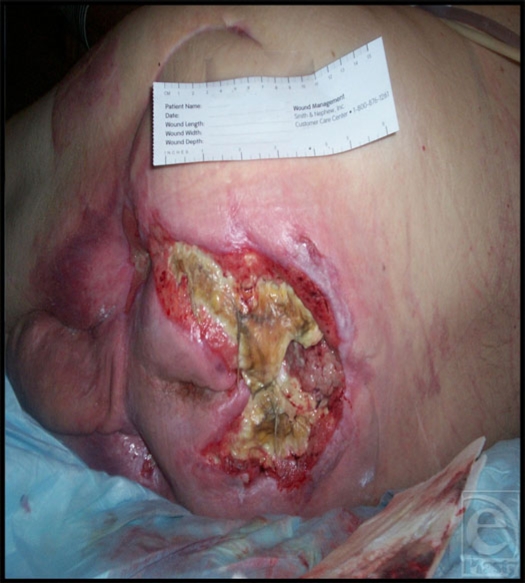
Large sacral ulcer before treatment with pulsed radio frequency energy.

**Figure 2:**
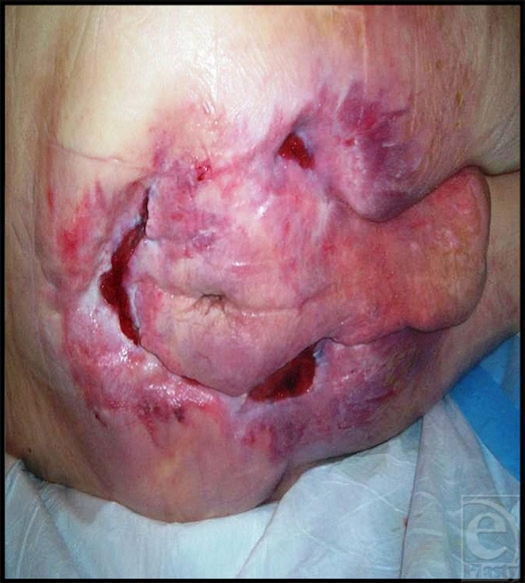
Sacral ulcer following 7 months of treatment with pulsed radio frequency energy with 88% reduction in surface area.

With twice-daily treatment, the 5-cm^2^ right lateral foot ulcer (stage III) healed to closure in 4 weeks, equating to a wound healing rate of 16.7 mm^2^/day; the left heel ulcer (stage III) decreased in size from 60 to 2 cm^2^ in 7 months (95% reduction; 28.5 mm^2^/day); and the large sacral ulcer (stage IV) (Fig 1) decreased from 295 to 20 cm^2^ in 7 months (88% reduction; 13.1 mm^2^/day; Fig 2), reaching full closure in 11 months.

## DISCUSSION

Pressure ulcers are caused by damage to the skin and soft tissue from pressure, friction, or shear force, especially in areas that lack subcutaneous tissue, such as the coccyx, hips, and heels. Nerve damage and loss of sensation from paralysis or diabetes, vascular disease, malnutrition, increased age, and smoking are all risk factors in the development of pressure ulcers. Patients with spinal cord injury are especially susceptible to developing pressure ulcers (50%–60%), many within the first 30 days after injury.^7^ Because they are unaware of evolving pathology, areas of damage enlarge quickly and can lead to cellulitis, infection, osteomyelitis, gangrene, and sepsis, especially if left untreated. Numerous treatment regimens and wound healing products are available but not one successful, clearly defined protocol exists.^8^ Despite the advances in traditional treatment and adjuvant therapies, some of these wounds do not close readily, and healing is still a lengthy, costly process.

Over the last 2 decades, our understanding of the cellular, molecular, and physiologic processes in wound healing has increased.^9^ The growth factors that stimulate cells required for tissue repair have been identified at a molecular level. In chronic wounds, these molecules are deficient, and signaling of fibroblasts and endothelial cells (among others) to proliferate and begin the healing process is interrupted.^10^

In 2002, George et al^5^ introduced a therapeutic approach that uses PRFE to endogenously stimulate growth factor production and incite mitosis in the wound bed. In in vitro studies, human and rat primary fibroblasts and epithelial cells were treated with PRFE for various time periods and at various doses with cellular proliferation assessed quantitatively by direct counting and spectrophotometric analysis 24 hours after treatment. Results were compared with serum-treated controls. The investigators found significantly increased proliferation versus control after one 30-minute PRFE treatment (*P* < .001). Furthermore, their results indicated that PRFE treatment induces growth factor production and stimulates cell replication through a calcium-mediated intracellular pathway. In another in vitro study, Gilbert et al^6^ reported that cell proliferation in human fibroblasts increased by up to 2-fold within 24 hours of PRFE treatment compared with sham-treated controls. The authors attributed cell proliferation to the activation of the p44/42 mitogen-activated protein kinase pathways by PRFE. The results of both studies indicate that PRFE treatment may be of value in the reparative process of chronic wounds.

Some studies have shown an average reduction of 30% (range 13%–48%) in ulcer surface area using traditional therapy in patients with stage II through IV ulcers compared with a 62% (range 37%–84%) reduction in 4 to 8 weeks in patients treated with various adjuvant therapies (ie, electrical stimulation, topical nerve growth factor).^11^–^14^ Our patient had 3 stage III and IV wounds that failed to heal with many years of traditional therapy. Using PRFE, the right lateral foot ulcer healed to closure in 4 weeks. The left heel ulcer reduced in size by 95% at 7 months after treatment and the large sacral ulcer reduced by 88% at 7 months and 100% in 11 months.

Given the marked beneficial effect in our patient who had 3 large wounds that were unresponsive to traditional treatment methods, PRFE with basic wound care, if administered early in the course of pressure ulcer therapy, might avoid the lengthy hospitalizations and repeated surgical procedures necessary for treatment of uncontrolled ulcers, reduce the need for antibiotics and pain medication, and decrease the overall cost of treatment for chronically ill or injured patients.
